# Topics of Public Interest

**Published:** 1906-04

**Authors:** 


### TOPICS OF PUBLIC INTEREST

### Unnecessary Blindness

[*Boston Evening Transcript.*]

In the February number of the New York State Journal of Medicine, Dr. F. Park Lewis, who is president of the State School for the Blind at Batavia, N. Y., has contributed an article on the prevention of unnecessary blindness. The most significant statement made by Dr. Lewis is, that of existing blindness, the cases in which the sight could certainly have been saved by treatment, average thirty-three per cent.; in thirty-nine per cent. of cases blindness might possibly have been avoided. This means that the absolutely unavoidable cases of blindness have been placed by specialists, whose word is authoritative, at twenty-eight per cent.

Such figures as these are striking, and raise conflicting emotions. It is terrible to think that seventy per cent. of the blind might possibly have retained their sight wholly or in part, if only the best knowledge of the present could have been utilized in their treatment. On the other hand, there is cause for great encour-

